# Special Issue “Structural Variations and Molecular Genetics of Hepatitis Virus and Related Viruses”

**DOI:** 10.3390/v13081456

**Published:** 2021-07-27

**Authors:** Kei Fujiwara

**Affiliations:** Department of Gastroenterology and Metabolism, Nagoya City University Graduate School of Medical Sciences, Nagoya 467-8601, Aichi, Japan; keifuji@med.nagoya-cu.ac.jp; Tel.: +81-52-853-8211; Fax: +81-52-852-0952

In this special issue, we present collected updated data on the hepatitis viruses. Articles on bioinformatics and molecular genetics illustrate the state-of-the-art of HBV research in particular. In addition, interesting data on HCV and HEV infection are included. 

Mathkar et al. [1] analyzed the HBV integration site using the VIcaller platform, which was developed to identify clonal viral integration in the human genome and to provide estimated integration allele frequencies [2]. The authors report their in-depth analyses of HBV integration events identified by VIcaller, in addition to characterization and pathway enrichment analyses of integration events. They found differences between tumor and paired normal tissues in nucleotide positions of the upper and lower breakpoints. The data showed that tumor tissues contained HBV enhancer or promoter regions, including part of the HBV X regions. In addition, they found that the lengths of inserted HBV sequences in tumor tissues were significantly shorter than in normal tissues. Further, they identified 48 novel HBV integration sites. Subsequent pathway enrichment analysis revealed multiple pathways including the alcohol dehydrogenase 4 gene pathway. They showed that the VIcaller platform provides detailed characterization of viral integration in cancer. 

Lin et al. [3] reviewed the role of pre-S gene deletion and pre-S deleted proteins in the development of hepatocellular carcinoma (HCC). They illustrated two patterns of ground glass hepatocytes (GGHs), namely, type I GGHs expressing globular or inclusion-like patterns of large surface proteins with deletions in the pre-S1 domain, and type II GGHs expressing large surface proteins with deletions in the pre-S2 domain. They further reported that the presence of type II GGHs was associated with shorter overall and recurrence-free survival of HCC patients. In addition, they explored the tumor immune microenvironment in HBV-related HCC. Activated pro-tumor regulatory T cells, lower levels of anti-tumor cytotoxic T cells, and higher levels of the immune checkpoint molecule programed death ligand 1 were observed in HCC patients with pre-S2 deletion. This suggests that pre-S2 deletion might act as a biomarker for poor responses to immune checkpoint inhibitors. 

Fujiwara [4] reviewed the new findings of genetic changes in HBV and hepadnaviruses, describing two patterns of structural variations (SVs). Complex SVs, composed of combinations of different SVs, were detected by studies of human HBV. Viral strains with such complex SVs potentially have a risk of causing severe liver disease by up-regulating of viral genome transcription, but further studies will be necessary to clarify their clinical roles. Other complexities are SV polymorphisms. Different orthohepadnaviruses possess unique SVs with those infecting different hosts having distinctive SVs. These SVs are conserved in viral groups, characterized by a different SV in the same position. The SV patterns separate certain genotypes or hosts and could be a key factor for detecting genetic diversity of hepadnaviruses.

Campos-Valdez et al. [5] summarized genetics and immunological research on HBV, describing the characteristics of HBV genotypes. In addition, they emphasized that most of the well-characterized of HBV genotypes are types A to D. We do not have enough information on HBV genotypes E to H which are endemic in low-to-medium-income countries where the disease might be sub-diagnosed. They mentioned that we also do not have enough information to determine whether HBV genotype H with core promoter mutations also increase the risk of HCC development.

Grigas et al. [6] reported that immunosuppressed individuals such as patients with inflammatory bowel disease (IBD) or solid organ transplant recipients had higher anti hepatitis E virus (HEV) IgG positivity than healthy individuals. The genetic sequence of an HEV strain recovered from one patient was analyzed, and identified as HEV genotype 3. That HEV strain showed high homology with those from wild boar. Further, HEV from the patient was co-cultured with several cell lines. Only MARC-45 cells (non-human origin) were observed to harbor HEV particles. The phylogenetic analysis revealed a close genetic relationship of this human HEV strain with wild animal and pig strains, suggesting possible cross-infectivity between human and animals in Lithuania.

Tung et al. [7] analyzed the prevalence of the HCV genotype (GT) 6 in Tainan city in Taiwan. HCV GT 6 is mainly observed in Southeast Asia and southern China. Previous reports showed that HCV GT 6 was rare in Taiwan. The authors found that the prevalence of HCV GT 6 in Tainan city was 17.1% (518/3022). They further determined that the frequency of HCV GT 6 infection in Tainan was higher in the area close to the two major rivers in the city. In addition, sequencing of core/E1 and nonstructural protein 5B revealed subtypes of HCV GT6. Two major subtypes of HCV GT6 in Tainan were 6g and 6w, which had a unique distribution.

In this special issue, the following questions were raised.

With the VIcaller method, comprehensive analysis of the HBV integrations was performed. Further studies are required to understand how HBV integration plays a role in HCC, for example, the meaning of the lengths of HBV integrates, the functional roles of inserted HBV DNA regions, the importance of the sequence orientation of insertions, and the proportions of cells with HBV integration.

Data have shown that pre-S deleted protein is associated with HCC development and recurrence. Additional studies are required to develop a new biomarker by using pre-S deletion and pre-S deleted protein. 

Novel genetic alterations in HBV designated complex structural variations (SVs) and SV polymorphisms have been clarified. However, we do not have sufficient information to fully characterize these genetic changes, and more genetic sequences and more data are required.

Features of HBV genotypes have been reported mainly in HBV genotypes A to D. We do not have sufficient information on HBV genotypes E to H.

A high prevalence of HEV in IBD patients and solid organ transplant recipients was documented. Further studies are required to show that zoonotic transmission is the cause of infection.

The existence of HCV GT6 was clarified in Tainan city in Taiwan. The study showed demographic backgrounds of subtypes 6g and 6w were different from those of subtype 6a, 6k, and 6n. Further studies should be performed to clarify the differences of treatment efficacy and incidence of hepatocellular carcinoma. In addition, the hypothesis that subtype 6g originated from indigenous people who lived on the ancient island of Taiwan should be clarified in an epidemiological study.

The above-listed issues point to the fact that genetic alterations of HBV, such as complex SVs, SV polymorphisms, genotypes, integrations, and pre-S deletions are important factors in chronic liver disease and in hepatocarcinogenesis. Fully comprehensive analyses of SVs and other genetic alterations are needed as the next step. In addition, epidemiological studies on HCV are required to clarify the origin of the virus. Furthermore, the clinical significance of chronic infection of HEV remains to be clarified

Concluding remarks. This Special Issue has focused on SVs in HBV, bioinformatic analysis, and the characteristics of hepatitis viruses. We thank all contributing authors. We hope that this Special Issue will help readers to take forward steps in hepatitis virus research.
